# Ophthalmic clinical officers: developments in Uganda

**Published:** 2014

**Authors:** Godfrey Kaggwa

**Affiliations:** Senior Ophthalmic Clinical Officer and outgoing President of the National Association of Ophthalmic Clinical Officers and Cataract Surgeons, Kampala City, Uganda. kaggwagodfrey@yahoo.com

**Figure:**
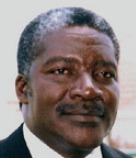
Godfrey Kaggwa

## Background

The population of Uganda is estimated to be over 33 million, with a prevalence of blindness of about 1%.

The following eye team members deliver eye care services at different levels.

- **Tertiary level:** ophthalmologists (there are about 40 in the country) and ophthalmic nurses (there are two), working at national and regional referral hospitals.
- **Secondary/district level:** ophthalmic clinical officers (OCOs). There are over 200 in the country.
- **Primary level:** nurses, midwives and other health workers who are trained in eye care and who work at health centres; they are known as primary eye care workers.
- **Community level:** community health workers (trained volunteers) who work in village health teams to provide health services at household level.

There is an existing referral system, with a good degree of teamwork, across all the levels.

**‘The majority of OCOs work in rural settings, where there is no ophthalmologist’**

Clinical officers or Uganda Registered Nurses (URN) can undergo a one-year diploma in ophthalmology (offered at the Jinja School for OCOs since 1989) in order to qualify as an OCO. The qualified OCO practices with the supervision of an ophthalmologist attached to the nearest regional referral hospital.

The majority of OCOs work in rural settings, where there is no ophthalmologist. According to the VISION 2020 targets, there should be ten OCOs or ophthalmic nurses per million population. This is not being achieved, however, as there are currently only 6.3 OCOs per million population.^1^ By 2020, Uganda will be even further away from meeting this target, as the population growth is exceeding the growth in the number of OCOs and mid-level personnel.^1^

## Core activities of ophthalmic clinical officers

In the rural areas, OCOs refer patients with complicated clinical problems to ophthalmologists and those requiring complicated refractions are referred to optometrists. They review patients after operations, perform extra-ocular surgery, assist surgeons in theatre, and conduct eye care outreach to schools, rural parts of the community and remote health facilities.

**Figure:**
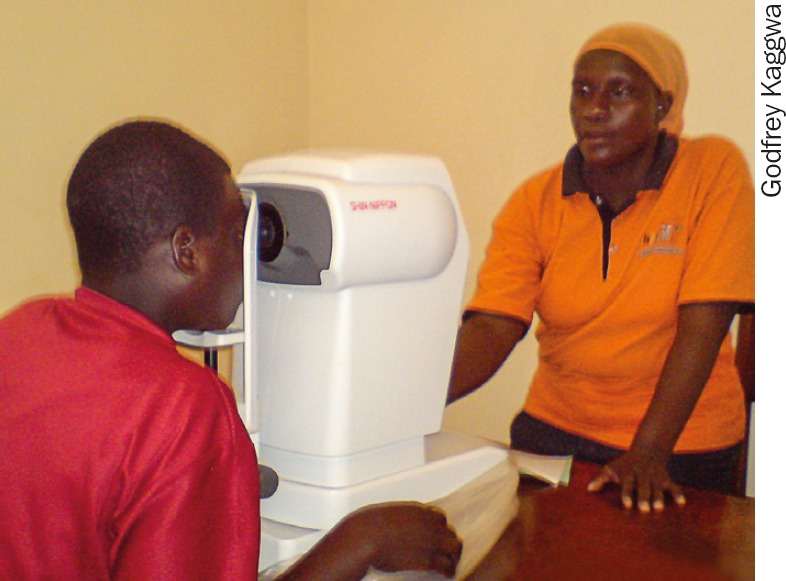
An OCO refractionist (right) with a patient

At district level, OCOs coordinate eye care services (including planning and budgeting) and also train and supervise other eye care personnel.

## Challenges

An OCO can become a senior and then a principal OCO. This is, however, only available to OCOs working at regional level. OCOs working at district level can be promoted within general medicine; however, they can then be sent to any health facility (sometimes to lead such a facility), where they will have very little time to practice ophthalmology.

OCOs can specialise as cataract surgeons, refractionists or low vision workers; however, these skills are not formally recognised within the existing government public service structure. Specialist OCOs therefore miss out on promotion and any accompanying salary increments.

Other challenges include:

- lack of funding for outreach activities and supervision (particularly for OCOs working in remote areas)
- ageing equipment
- lack of funding and opportunities for continued professional development.

## New developments

OCOs in Uganda are organised in a registered association called the National Association of Ophthalmic Clinical Officer and Cataract Surgeons (NAOCOCS). The association addresses coordination, governance and the social concerns of OCOs. The association organises annual continuing professional development workshops which most eye care workers attend. OCOs are represented at the National Prevention of Blindness Committee and its technical arm at the Ministry of Health.

NAOCOCS has identified the following priorities for action:

- creation and establishment of posts for senior and principal OCOs at district level
- promotion or better remuneration for OCOs who have qualified as cataract surgeons, refractionists or low vision therapists
- a review of the OCO training curriculum to ensure it meets international and regional standards and is relevant to each country's current eye care needs
- improving community involvement in eye care services
- improving eye care data collection, reporting, analysis and utilisation at all levels.

**OCOs in Africa**

**Ingrid Mason**

CBM Regional Director Central Africa

Ophthalmic clinical officers (OCOs) were introduced into East and Central African health worker government schemes in the mid-1980s. Given the desperate shortage of ophthalmologists, their main purpose was to identify and treat patients with common eye conditions. A similar cadre, the Technologiste Superior d'Ophthalmologie (TSO) was later introduced in several Francophone countries in West Africa for much the same reason.

OCOs and TSOs remain the backbone of the eye health workforce in many countries in East, Central and West Africa. They are primarily deployed in secondary health care units. They see patients referred from the community and refer patients with complicated problems to the tertiary setting.

In the district or secondary health care setting, OCOs and TSOs work alongside the other eye health workers such as ophthalmic nurses, optometrists, refractionists and low vision therapists as a comprehensive eye health team. In many countries, the OCO and TSO remains a valuable team member who is able to independently manage many eye conditions.
